# From fibres to adhesives: evolution of spider capture threads from web anchors by radical changes in silk gland function

**DOI:** 10.1098/rsif.2024.0123

**Published:** 2024-07-31

**Authors:** Jonas O. Wolff, Leah J. Ashley, Clemens Schmitt, Celine Heu, Denitza Denkova, Maitry Jani, Veronika Řezáčová, Sean J. Blamires, Stanislav N. Gorb, Jessica Garb, Sara L. Goodacre, Milan Řezáč

**Affiliations:** ^1^ Evolutionary Biomechanics, Zoological Institute and Museum, University of Greifswald, Loitzer Str. 26, Greifswald 17489, Germany; ^2^ School of Natural Sciences, Macquarie University, Sydney, New South Wales 2109, Australia; ^3^ ARC Centre of Excellence for Nanoscale BioPhotonics (CNBP), Department of Physics and Astronomy, Macquarie University, Sydney, New South Wales 2109, Australia; ^4^ School of Life Sciences, University of Nottingham, University Park, Nottingham NG7 2RD, UK; ^5^ Department of Biomaterials, Max Planck Institute of Colloids and Interfaces, Am Mühlenberg 1 Potsdam 14476, Germany; ^6^ Katharina Gaus Light Microscopy Facility (KGLMF), Mark Wainwright Analytical Centre, University of New South Wales, UNSW Sydney NSW 2052, Australia; ^7^ Evolution and Ecology Research Centre, School of Biology, Earth and Environmental Sciences, University of New South Wales, UNSW Sydney NSW 2052, Australia; ^8^ ICFO—Institut de Ciencies Fotoniques, The Barcelona Institute of Science and Technology, Castelldefels (Barcelona) 08860, Spain; ^9^ Functional Biodiversity Team, Crop Research Institute, Drnovská 507, CZ-16106 Prague 6 – Ruzyně, Czechia; ^10^ Functional Morphology and Biomechanics, Zoological Institute, University of Kiel, Am Botanischen Garten 1-9 Kiel, 24098, Germany; ^11^ Department of Biological Sciences, University of Massachusetts Lowell, Lowell, MA 01854, USA

**Keywords:** spider silk, piriform silk, adhesive, spidroin, viscid silk, convergence

## Abstract

Spider webs that serve as snares are one of the most fascinating and abundant type of animal architectures. In many cases they include an adhesive coating of silk lines—so-called viscid silk—for prey capture. The evolutionary switch from silk secretions forming solid fibres to soft aqueous adhesives remains an open question in the understanding of spider silk evolution. Here we functionally and chemically characterized the secretions of two types of silk glands and their behavioural use in the cellar spider, *Pholcus phalangioides.* Both being derived from the same ancestral gland type that produces fibres with a solidifying glue coat, the two types produce respectively a quickly solidifying glue applied in thread anchorages and prey wraps, or a permanently tacky glue deployed in snares. We found that the latter is characterized by a high concentration of organic salts and reduced spidroin content, showing up a possible pathway for the evolution of viscid properties by hygroscopic-salt-mediated hydration of solidifying adhesives. Understanding the underlying molecular basis for such radical switches in material properties not only helps to better understand the evolutionary origins and versatility of ecologically impactful spider web architectures, but also informs the bioengineering of spider silk-based products with tailored properties.

## 1. Background

Spiders are one of the most successful and dominant groups of invertebrate predators in terrestrial ecosystems [1]. This success is the result of the evolution of a unique material—spider silk—and its versatile use in complex architectures, such as egg cases, retreats and webs. Spider webs exhibit an enormous diversity of shapes and functions. One of the ecologically most influential classes of webs are aerial snares, such as orb webs and cob webs, that capture a broad range of insects [2–4]. Most such webs rely on an adhesive coating of silk lines that retain the stopped prey, allowing the spider to subdue and safely handle the prey. It has been proposed that viscid silk has been a key innovation in spider evolution that has led to a boost in speciation and the expansion into new niches [5–7]. However, the evolutionary origin of viscid silk remains an open question [8].

The investigation of convergent evolution of viscid silk could shed light on this unresolved problem. While most viscid silk users belong to the large clade of orb-web and cobweb spiders (Araneoidea), it is a poorly known fact that many daddy-longleg spiders (Pholcidae), including the cosmopolitan house spider *Pholcus phalangioides*, use similar silks [9]. Prey capture behaviour in *P. phalangioides* exhibits a number of striking similarities with that of very distantly related cobweb spiders (basal branch of Theridiidae, which includes species such as the black widows, *Latrodectus* spp.). Both groups build three-dimensional space webs, which are composed of an aerial tangle and vertical lines, some of which may be coated with a strip of viscid silk close to the substrate—so-called gumfoot threads [10–12]. While the aerial tangle may stop small flying insects, the gumfoot threads are used to trap running prey, such as ants. Such threads are spring-loaded [13] and attached with a special weakened silk anchor that easily detaches upon contact [14,15], lifting the stuck prey into the web. These spiders hang from the web in an inverted position and reel in gumfoot lines with entrapped prey [10]. In addition, both pholcids and theridiids immobilize prey by throwing sticky lines on it [16–19]. These lines are pulled from the spinnerets with the hind legs, while the spider is hanging above the prey or holding it with the tips of its middle legs [20]. Using this technique, these spiders can subdue prey much larger and stronger than themselves, such as other spiders and even small vertebrates [18,20–22]. This offers them an enormous spectrum of prey sizes, which may be one of the reasons why these spiders are so abundant and cope well with extreme environments such as the interiors of urban buildings.

Thus far, almost all of our knowledge on the chemistry and mechanical function of viscid silk comes from studies on representatives of the Araneidae and Theridiidae. In these families, viscid silk is produced by the aggregate silk glands with spigots on the posterior lateral spinnerets [23]. The aggregate secretion contains specific silk proteins called aggregate spidroins (AgSp1 and AgSp2) [24,25], carbohydrates [26] and a high concentration of organic and inorganic salts [9,27,28]. This aqueous solution exhibits the characteristics of viscoelastic fluids in the Theridiidae [29] and of viscoelastic solids in Araneidae [30], and self-organizes into beads-on-a-string structures after its application onto the silk line. Adhesion is recruited by the glycosylated, highly polar and very stretchy AgSp2 [24], aided by the hygroscopic salts that keep it hydrated, even in dry environments, and facilitate its interaction with the prey surface [28].

Pholcids do not possess aggregate glands, but show some peculiar silk glands with widened openings, which have been called ‘mucous glands’ [31–33]. These glands have been hypothesized to be strongly modified piriform glands because they are located in the same (homologous) position as the piriform glands in other spiders, terminating on the anterior lateral spinnerets [34]. Piriform glands are present in nearly all araneomorph spiders and usually fulfil a stereotypic function: the attachment of silk threads to substrates and the fastening of thread junctions—important prerequisites to build complex three-dimensional architectures from one-dimensional materials [35]. The secretion of the piriform glands is usually biphasic and consists of a central fibre and a filamentous glue coat, each produced by specialized secretory tissue and simultaneously extruded from the gland [36,37]. After extrusion the initially fluid glue coat rapidly solidifies and forms a stable bond [38]. Web anchors of *P. phalangioides* reflect the modification of piriform glands into ‘mucous glands’: they are very distinct from those of other spiders by representing a large puddle of glue instead of the typical numerous submicron-sized glue trails [31,35,39,40]. We hypothesized that these modifications represent an evolutionary pathway to the evolution of viscid silk mediated by a functional expansion of piriform silk (i.e. its use in sticky wrap attacks in addition to the basal thread anchor function) and by a differentiation of the piriform gland array into two types.

To test this hypothesis, we performed an analytical study of *P. phalangioides*' glue products. We analysed the structure of the glue-coated lines and the chemical profiles of the glue in their silk anchors, wrapping threads and gumfooted lines, and the protein expression patterns of the glue glands. This was combined with an anatomical study of the spinning apparatus and the detailed observation of silk spinning behaviour. We aimed to answer: (i) which glands are involved into the production of different silk and glue products, (ii) how the contrasting properties of different glue products of *P. phalangioides* correlate with differences in chemical composition and spidroin expression patterns, and (iii) if glues are combined with different fibrous products to tailor the function-specific material properties.

## 2. Material and methods

### 2.1. Animals and silk sampling

*Pholcus phalangioides* were collected in university and residential buildings and kept under normal room conditions (details in S1). Gumfoot and wrapping lines were collected on coverslips (for light microscopy and atomic force microscopy (AFM)) or quartz slides (for Raman spectroscopy) and stored in dry plastic boxes. Gumfoots were collected from webs by touching them with the slide, which resulted in viscid silk becoming attached to the slide and the thread breaking off the web. The sticky wrapping lines were collected by feeding the spider with a cricket nymph (*Acheta domesticus*) and holding the slide between the spider and the prey just after the spider started wrapping and pulling up the prey into its web. Silk anchors and some gumfooted lines were collected by placing glass or quartz slides into the terrarium overnight, so that spiders attached some lines to the slides during web construction.

Exemplary webs and wrap attacks were photographed and filmed with a Canon EOS 300 DSLR (Canon Inc., Tokyo, Japan).

### 2.2. Light and electron microscopy

The structure of silk products was studied with conventional and phase contrast transmission light microscopy using Leica M205 A and Leica DM5000B stereo microscopes (Leica Microsystems GmbH, Wetzlar, Germany), and an AXIO Observer.A1 transmission light microscope (Carl Zeiss AG, Oberkochen, Germany). Samples on glass cover slips were imaged in air in the native state and after scratching with an insect pin to split and separate bundled fibres. Contrast and sharpness of digital photographs were enhanced in ImageJ 1.5 [41]. Images were analysed for the glue structure and the presence of embedded fibres with different diameters.

The silk glands of freshly killed spiders were dissected in embryo dishes using physiological solution (0.9% aqueous solution of sodium chloride) and viewed under an Olympus SZX12 stereomicroscope (for details see [42]). They were subsequently transferred in a drop of physiological solution onto a microscope glass slide with a small prefabricated circular impression and photographed under a Nikon Eclipse 80i light microscope. Silk glands of adult females and males as well as juveniles were studied. No apparent differences in the presence or relative size of individual glands were found between males and females or ontogenetic stages. Adult females were used for photographic documentation because of their larger size.

Native spinnerets and spigots were observed in one female with cryo-scanning electron microscopy following the methods outlined in Wolff *et al*. [38]. In order to handle the spider in the chamber of the SEM, all legs were removed after snap freezing in liquid nitrogen.

Silk specimens were air dried, mounted on stubs with double-sided carbon tape, sputter coated with Au-Pd and viewed in a Zeiss EVO LS10 scanning electron microscope (Carl Zeiss AG, Oberkochen, Germany).

### 2.3. High-speed videography

The production of dragline anchors was observed with high-speed videography to identify the spigots involved in the production of anchor and dragline. Spinning events were filmed from underneath a glass slide, using an inverted reflection interference contrast microscope (AXIO Observer.A1, Carl Zeiss AG, Oberkochen, Germany) with an attached Photron Fastcam SA 1.1 high-speed video camera (Photron Inc., San Diego, CA, USA) at a frame rate of 1000 fps, as described in Wolff *et al*. [38]. Additional observations were made with a Basler Ace 640 × 480pix USB 3.0 high-speed video camera (Basler AG, Ahrensburg, Germany), equipped with a Navitar Precise Eye extension tube including a 1.33× magnification lens (Navitar, Inc., Rochester, NY, USA), following the methods outlined in Wolff [43] and using frame rates of 500 fps. In total, 30 spinning events were observed in four females and one male.

### 2.4. Atomic force microscopy

Each of the two fresh samples of dragline anchors and gumfoot lines (collected on the day of testing) were studied with a Bruker BioScope Resolve atomic force microscope (Bruker, Billerica, MA, USA), and the data were acquired with the Bruker NanoScope Analysis 2.0 software as described in Blamires *et al*. [44]. The only image corrections applied to the AFM images were flattening of the topography and contrast adjustment to facilitate visualization. We used Bruker RTESPA-525 silicon probes with nominal tip radius 8 nm, resonance frequency 375–675 kHz and spring constant 100–400 N m^−1^. The cantilevers were calibrated as described by Heu *et al*. [45] (details in electronic supplementary material, S1). A new probe was used for each sample to avoid the potential effect of tip contamination with adhesive fluids from the silks.

Different areas (with and without glue) of the samples were deposited on glass and scanned in air using Bruker's proprietary PeakForce Tapping mode, ScanAsyst and the PeakForce quantitative nanomechanical property mapping (QNM) capability [46] (details in electronic supplementary material, S1).

Adhesion values were calculated by measuring the difference between the base line and the minimum peak of the retraction curve.

### 2.5. Raman spectroscopy

Raman spectra were acquired with a confocal Raman microscope (CRM200, WITec, Germany) equipped with a piezo-scanner (P-500, Physik Instrumente, Karlsruhe, Germany). The diode-pumped near infrared laser (*λ* = 785 nm, Toptica Photonics AG, Gräefelfing, Germany) was focused on the sample through a 50× objective (Nikon, NA = 0.65). The laser power on sample was set to 10 mW. The spectra were acquired using an air-cooled CCD (DU401A-DR-DD, Andor, Belfast, North Ireland) behind a 300 g mm^−1^ grating spectrograph (Acton, Princeton Instruments Inc., Trenton, NJ, USA) with a spectral resolution of 6 cm^−1^. The ScanCtrlSpectroscopyPlus software (v. 1.38, WITec, Ulm, Germany) was used for measurement set-up. At least five single measurements with 10 accumulations each and an acquisition time of 10 s for each accumulation were acquired from different points within the respective materials and averaged. Betaine (trimethylglycine, Sigma) was used as a spectral standard, as it was previously found to be a major compound of the water soluble fraction of pholcid gumfoot glue [9]. Raman spectra of glues were taken from clear glue regions devoid of embedded fibres.

WITec Project FIVE (Version 5.2.4.81, WITec, Ulm, Germany) was used for data analysis. Spectra were background subtracted and normalized to the spectral region between 2800 and 3100 cm^−1^, assuming a similar density of materials and a random distribution of CH-bonds in the excitation volume of the respective measurements.

### 2.6. Transcriptomics

In short, de novo transcriptomes were generated from separated large piriform glands, and small piriform glands, of three female *P. phalangiodes* using Illumina RNA-Seq. A full description of the transcriptomics and bioinformatic methods is provided in electronic supplementary material, S1. Transcriptomes of *P. phalangioides* whole abdomens and dissected total array of silk glands were taken from a previous study [47]. All transcriptomes were BLAST searched for spidroin transcripts against the nr NCBI and a custom-built reference database. The whole abdomen tissue transcriptome was used as a reference to compare relative expression across all tissue types. To clarify the relationship of *Pholcus* silk genes with functionally annotated spidroins from the literature, a phylogenetic analysis of the spidroin C- and N-terminal domains was performed, using maximum likelihood in RAxML 8.2.12 and Bayesian phylogenetic inference in Mr Bayes v. 3.2.7.

#### 2.6.1. Silk type abbreviations

Ac, aciniform gland silk; Lpi, large piriform gland silk; MA, major ampullate gland silk; miA, minor ampullate silk; sPi, small piriform gland silk.

## 3. Results

### 3.1. Silk gland morphology

**Figure 1.**
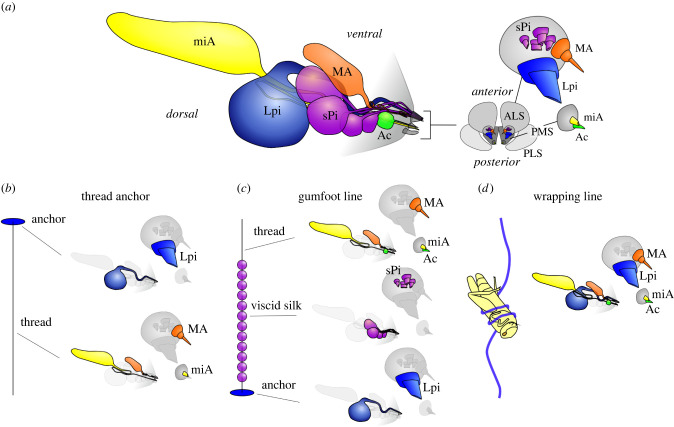
Schematic drawing of spinning apparatus of *P. phalangioides* and summary of the glandular origin of its different silk products. (*a*) Spinning apparatus comprised silk glands (left; glandular set of one body half shown in lateral view) and spinnerets (middle; modified appendages bearing the spigots, ventral view with anterior part on top) and spigots (nozzle-like protuberances carrying the gland duct openings highlighted on magnified spinneret tips in the right, apical view). The PLS does not bear any spigots in this species. For SPi glands and spigots only four are shown here for the sake of simplicity. (*b*) Proposed glandular origin of conventional thread (e.g. dragline) anchors after high-speed video recording observations of spinning events. Note that both miA and MA fibres are usually only present close to the anchor and MA fibres are often discontinued in the following (depending on drawing speed). (*c*) Proposed glandular origin of different parts of gumfoot lines after microscopical observations and comparative Raman spectroscopy. (*d*) Proposed glandular origin of sticky wrapping lines after microscopical observations and comparative Raman spectroscopy. miA fibres are usually not included in the initial stage of the wrapping, when sticky silk is used, but often become involved into later stages of the wrapping. Ac, aciniform gland/spigot; ALS, anterior lateral spinneret; Lpi, large piriform gland/spigot; MA, major ampullate gland/spigot; miA, minor ampullate gland/spigot; PLS, posterior lateral spinneret; PMS, posterior median spinneret; sPi, small piriform glands/spigots.

The spinning apparatus of *P. phalangioides* comprises one pair of major ampullate glands, one pair of minor ampullate glands, one pair of aciniform glands, one pair of large glue glands and six pairs of small glue glands (figure 1; electronic supplementary material, S2). Ampullate and aciniform glands are of a simple structure (i.e. sac-like reservoir, no tail regions; electronic supplementary material, S2.B,C) and homologized by their shape and the specific placements on the spinnerets (see Eberhard [48] for details). A peculiarity of *Pholcus*, in complete contrast to other spiders, is that the major ampullate glands are considerably smaller than the minor ampullate glands, along with a difference in the diameters of the spigot openings and, accordingly, fibres. The shape of the glue glands and the spatial placement of their spigots on the anterior lateral spinnerets resemble those of the piriform glands in other spiders, which satisfies homology criteria. However, the enormous size of those glands, their differentiation into large (electronic supplementary material, S2.E) and small piriform glands (electronic supplementary material, S2.F), and the structure of their spigots are characteristics specific to Pholcidae. The spigots of the piriform glands are shortened and widened, and have slit-like openings, suited to expel larger amounts of secretion. The spigots of the small glue glands are only half the size of the large glue glands, but otherwise have a similar structure.

### 3.2. Structure of pholcus glue products

#### 3.2.1. Gumfoots

**Figure 2.**
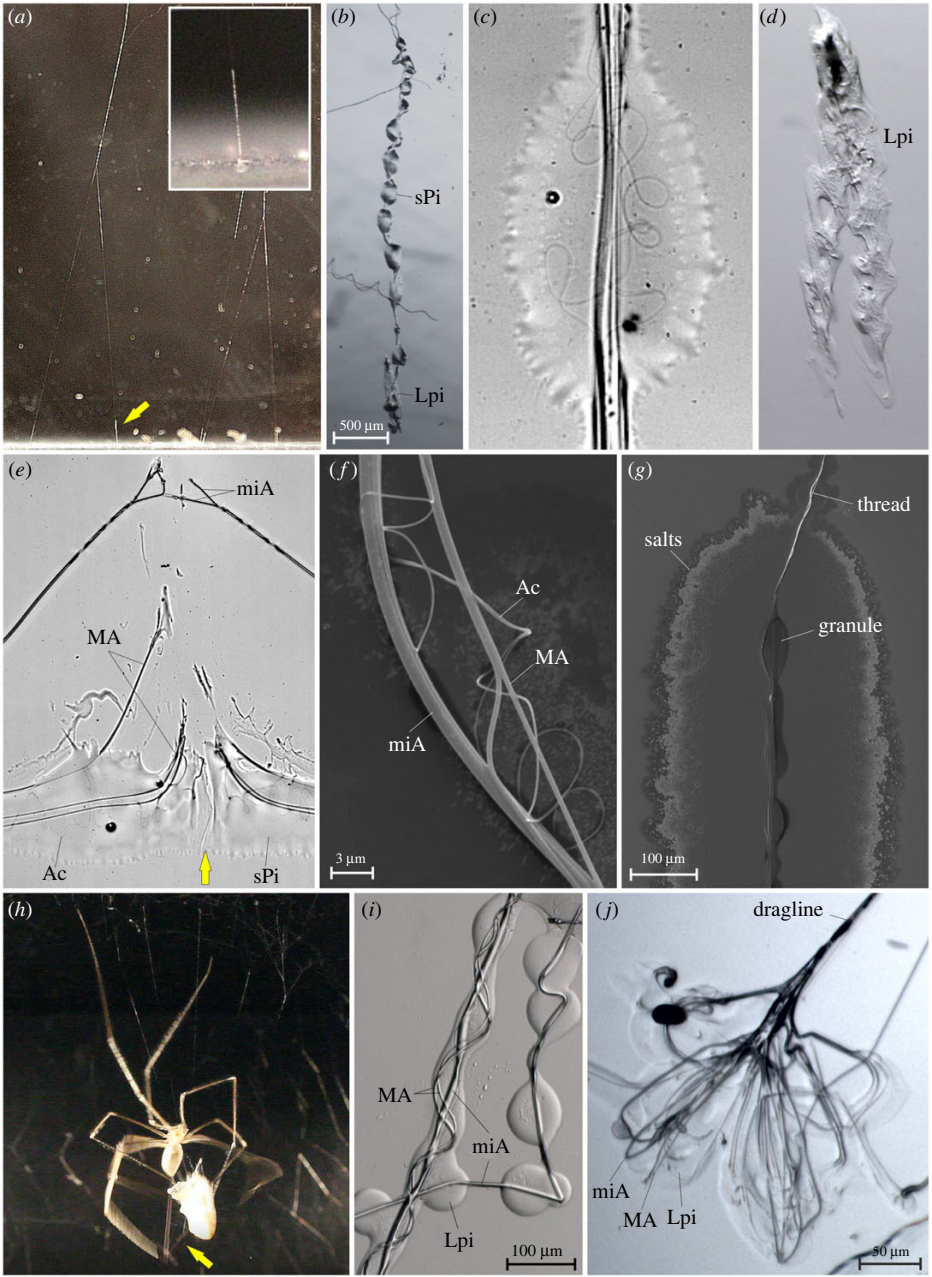
*Pholcus phalangioides* produce both solidifying and vicid silk adhesives, with different functions. (*a*) Detail of web, showing gumfooted lines (a single gumfoot is highlighted by the yellow arrow and shown magnified in the inset). (*b*) Detail of a gumfoot on glass slide, including anchorage (bottom). (*c*) Detail of viscid silk droplet from gumfoot. Note the inclusion of thin, looped silk fibres. (*d*) Thread anchor of a gumfoot line. Note reduced structure compared with the regular dragline anchor (shown in *g*). (*e*) Detail of scratched gumfoot line (direction of scratch indicated with yellow arrow), showing the composition of the axial fibre bundle (2 miA + 4 MA fibres). Thin curled fibres are probably Ac silk. (*f*) Scanning electron microscropy (SEM) image of the axial line of a gumfoot thread, showing thick (miA), medium (MA) and thin (Ac) fibres. (*g*) SEM image of remnants of viscid silk droplets of gumfoot thread. Crystals deposited at the edges of the glue droplet are hypothesized to indicate organic salts and the core granule wrapped around the axial line to be the proteinaceous (silk) deposit of the sPi secretion. (*h*) Video still of a wrap attack onto a cricket. The yellow arrow points to the paired sticky wrapping line that is handled with the hind legs. (*i*) Detail of wrapping line. (*j*) Detail of dragline anchor. Ac, aciniform gland silk; Lpi, large piriform gland secretion; MA, major ampullate gland silk; miA, minor ampullate gland silk; sPi, small piriform gland secretion.

Gumfooted lines were built by the spiders between the web sheet and the bottom substrate only if the supporting structure (e.g. box walls) had the correct dimensions. The length of gumfooted lines usually did not exceed 10 cm, and if the spider was kept in an enclosure higher than 15 cm, it often built a web that only consisted of the horizontal or dome-like sheet and regular anchor lines. Gumfoot threads were more or less vertical fibre bundles that bifurcated closer to the substrate, with each branch being connected to the ground substrate and bearing a viscous mass along 2–5 mm of its lower end (figure 2*a*). The viscous mass often formed regular beads-on-a-string structures, however, lacking the smaller satellite droplets characteristic for the viscid silk of araneoids (electronic supplementary material, figure S3.B). The thread was a bundle of four median-sized and two thick fibres (figure 2*e,f*). In addition, there were two nano-fibres forming loops and curls in the droplet (figure 2*c,e,f*).

#### 3.2.2. Wrap attack silk

The glue-coated lines used in wrap attacks (figure 2*h*) included the median-sized fibres and, occasionally, thick fibres, which were either bundled or separate. To these varying amounts of a solidifying glue were attached. The glue may form a cylindrical coat or droplets (as in figure 2*i*) along the fibres. The glue sometimes formed larger puddles with the embedded fibres exhibiting a swollen shape. This varying structure might be due to the fast flow during the rapid pulling of the silk with the hind legs and the in-liquid spinning of the fibres. At later stages of the wrapping less glue was used and the thick and medium-sized fibre types were more frequently included.

#### 3.2.3. Silk anchors

Dragline and web anchors were bi-symmetrical structures, comprising looping glue trails with median and thick fibre types embedded (figure 2*j*). Both fibre types usually followed the glue trails; however, the thick type sometimes formed additional curls in the median part of the structure and was sometimes absent from the lateral glue trails. All fibres were combined in a bundled thread leaving the glue mass. The anchors of gumfoot threads had a different structure. These consisted only of a simple strip-like glue deposit and did not appear to have any embedded fibres (figure 2*d*). The thread was only weakly attached to this glue mass and broke off very easily upon contact.

**Figure 3.**
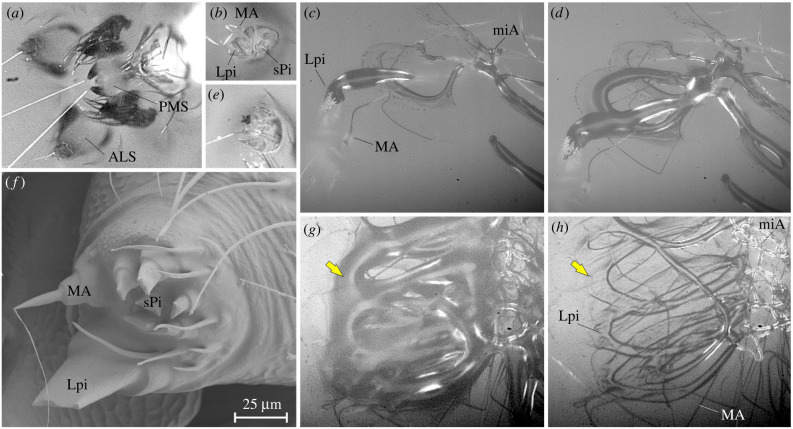
Solidifying glue is spun into dragline anchors. (*a*) Video still of spinning apparatus with a dragline. Each a fibre is contributed by the MA gland of the ALS and the miA gland of the PMS. (*b,e*) Magnified details of ALS with fibre emerging from the MA spigot. (*c,d,g,h*) High-speed video stills from reflection interference contrast microscopy of anchor spinning events (filmed at 1000 frames per second). (*c,d*) Two stills from the same sequence, highlighting the action of Lpi, MA and miA glands. (*g,h*) Two stills from the same sequence, separated by 1.2 s, directly after spinning. Note the change in optical properties of the secretion caused by glue crystallization (e.g. arrow). (*f*) Cryo scanning electron microscopy image of an ALS showing the native state of spigots. The highly enlarged piriform gland spigots of pholcids have a flexible wall with transversal ribs and slit-like openings that are closed in rest. The surrounding cuticule is visibly coated with a fluid that may be the previously reported [49] hydrocarbon coat that prevents adhesion of the secretions to the own body. ALS, anterior lateral spinneret; Lpi, large piriform gland spigot/secretion; MA, major ampullate gland spigot/silk; miA, minor ampullate gland spigot/silk; PMS, posterior median spinneret; sPi, small piriform gland secretion.

The observation of dragline anchor spinning events with means of high-speed videography revealed that the glue used herein solely emerged from the single large piriform gland spigots. The median fibre type originated from the major ampullate gland spigots and the thick fibre type from the minor ampullate gland spigots (figure 3*a–e*). The glue solidified within about 1 s after its extrusion, as indicated by a change in optical properties (figure 3*g,h*). The minor ampullate silk was directly ejected into the glue mass, before it dried. In contrast to the piriform silk of other spiders, the piriform secretions of *P. phalangioides* did not contain a visible fibre core (e.g. figure 3*c,d,g,h*).

The origin of gumfoot and wrap attack glues was inferred from comparing their chemical profiles (see §3.4) with that of the silk anchor glue, because the emergence of silks could not be filmed in close-up. Based on the fine structure of the curled nano-fibres embedded in the gumfoot glue we interpreted those as products of the (not observed in action) aciniform glands, whose spigot pore opening matched the thin diameter of these fibres. Our conclusions on glandular origins of different silk products are summarized in figure 1*b–d*.

### 3.3. Surface profiles and mechanical properties of pholcus glue products

**Figure 4.**
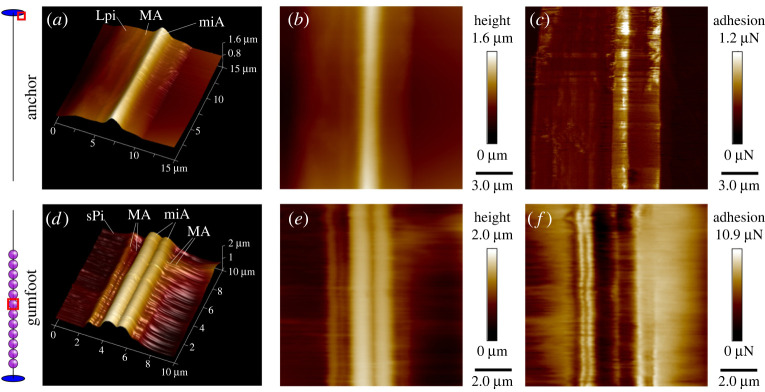
Nanoscale topography and mechanical properties differ between solidifying and viscid piriform silks in *P. phalangioides*. Peak force QNM mapping of a section of a dragline anchor (*a–c*) and a gumfoot thread (*d–f*). (*a,d*) Three-dimensional view of surface profile. (*b,e*) Height map. (*c,f*) Adhesion map. Lpi, secretion of large piriform gland; MA, silk fibre of major ampullate gland; miA, silk fibre of minor ampullate gland; sPi, secretion of small piriform gland.

AFM of a section of a dragline anchor (figure 4*a–c*) and a gumfoot thread (figure 4*d–f*) confirmed the inclusion of minor ampullate fibres (miA) and major ampullate fibres (MA). In particular, each glue trail in the thread anchors (figure 4*a–d*) contained 1 MA + 1 miA fibres, while the gumfoot lines were composed of 4 MA + 2 miA. As expected, Peak Force adhesion maps show that the solid anchor glue exhibits stronger adhesion than glass due to its higher softness (figure 4*c*). The surface of the gumfoot glue could not be clearly imaged due to its fluidic and adhesive properties. We found that the adhesion of the gumfoot glue was 10 to 20 times higher than that of the (solidified) anchor glue (figure 4*f*). Adhesion was substantially lower on top of the embedded miA fibres than the flanking droplet regions (figure 4). This suggests, that these fibres do not contribute to the adhesiveness of the gumfoot, but rather have the function to stabilize the structure of the gumfoot. Maps of the anchor showed an opposite trend (figure 4*c*): adhesion of the glue layer was higher on top of the embedded miA fibre than on the glass substrate, indicating an enhancing effect of the fibre, presumably by adding softness to the material. For the interpretation of the adhesion maps in figure 4*c,f*, it is important to note that only values on the flat top fibre surface (fibre ‘spine’) are comparable as the contact area between the cantilever tip and the sample is higher on the fibres flanks [50].

### 3.4. Comparison of chemical profiles of different glue products

**Figure 5.**
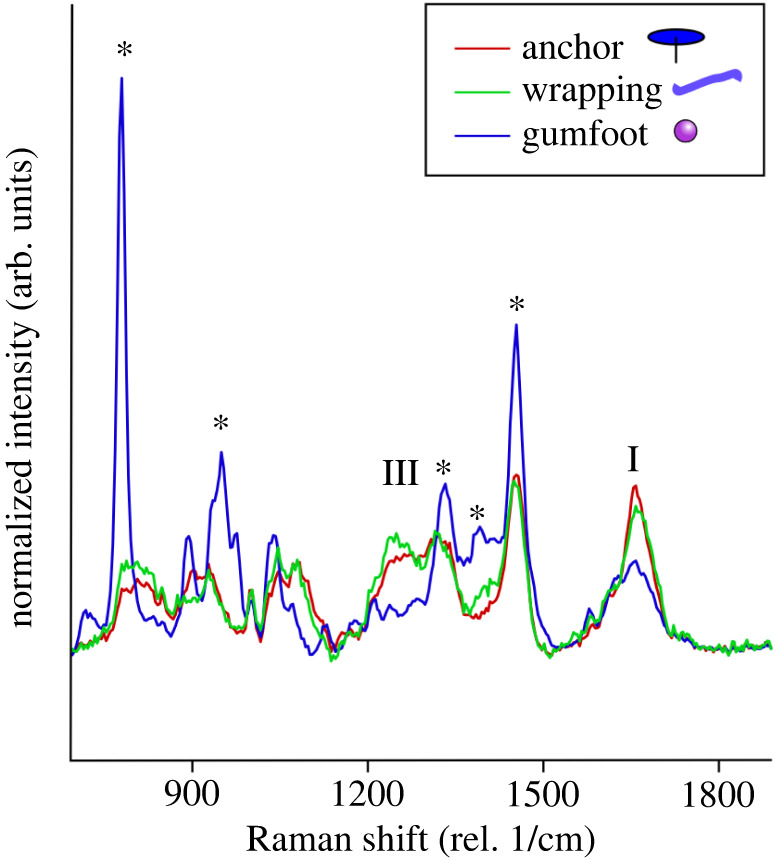
Chemical profiles indicate that *Pholcus* solidifying and viscid glues differ in the mix of proteins and organic salts. Averaged Raman spectra for the glue fractions of dragline anchor (red line), wrapping thread (green line) and gumfoot thread (blue line) samples on quartz. Asterisks indicate peaks that are characteristic for betaine (trimethylglycine). Roman numbers indicate peaks characteristic for proteins (I, amide I band; III, amide III band; after Lefèvre *et al*. [51]). See electronic supplementary material, S4 for full spectra.

The Raman spectra of glues from dragline anchors and wrapping threads were similar (figure 5, red and green lines), indicating that both may be products of the same type of gland. The spectrum contained peaks characteristic of large proteins (i.e. amide I and III bands, annotated after Lefèvre *et al*. [51]). By contrast, the viscid glue of gumfoot threads showed a highly aberrant profile (figure 5, blue line). The spectrum resembled that of betaine (trimethylglycine) (after Jehlička *et al*. [52]; electronic supplementary material, figure S4). In addition, a broad peak in the region of the amide I band was present, but peaks in the region of the amide III band were only weakly present.

### 3.5. Gland-specific spidroin expression patterns

We assembled transcriptomes from *P. phalangiodes* tissues, including from separated large and small piriform silk glands (electronic supplementary material, table S1.1). From these transcriptomes we identified six spidroins (variants 1–6) with unique terminal domains and distinctive repetitive sequence, suggesting each is encoded by a separate gene (electronic supplementary material, S5). We compared the expression of spidroins 1–6 and found that spidroins 1, 3, 5 and 6 exhibited elevated expression in the large piriform glands (table 1). Variants 2 and 4 exhibited no or very low expression in the piriform glands. However, variant 2 was the highest expressed across total silk glands and probably is a spidroin specific to the ampullate glands. The repetitive regions of variants 2 and 4 are similar to araneoid MaSp repetitive sequences, with both being glycine rich, and variant 2 containing poly-alanine motifs, whereas variant 4 contains GPGG repeat motifs (electronic supplementary material, S5). Two spidroins specific to the large piriform glands (variants 5 and 6) exhibited spidroin typical N-termini, but had missing or modified C-termini (electronic supplementary material, S5).

**Table 1.** List of spidroin sequences (with Genbank accessions where the terminal domain is identical to deposited sequence) assembled from the transcriptome of *P. phalangioides*, with relative transcription levels (TPM) per tissue against base transcription level (WA). When interpreting these numbers it should be kept in mind that each transcriptome assembly was a pool of tissues from different groups of spiders. Lpi, large piriform gland; sPi, small piriform gland; TSG, total (all) silk glands; WA, whole abdomen.

Putative spidroin (aggregated sequences)	Lpi_v_WA	sPi_v_WA	TSG_v_WA	WA_v_WA	Lpi/TSG
AVH80551.1 spidroin variant 1	4076.185	0	7633.224	182.642	0.534
AVH80550.1 spidroin variant 2	0	0	17 225.910	14 742.985	0
AVH80548.1 spidroin variant 3	3359.488	0.020	6147.211	288.191	0.547
AVH80547.1 spidroin variant 4	0.177	0.175	1622.254	466.890	0
spidroin variant 5 missing Cterm	3508.178	0	1654.995	98.471	2.120
spidroin variant 6 with modified Cterm	1641.543	0	496.145	12.340	3.309

Transcriptomes of the large and small piriform glands were highly contrasting. Except for spidroin variant 2, all spidroins identified were found to be present in the large piriform glands. However, the small piriform glands only exhibited very low levels of variant 3 and 4. Ninety of the 100 transcripts with highest expression in the large piriform library had BLASTx matches against nr (at e = 10); whereas only 60 of the top 100 expressed transcripts in the small piriform gland library had similar BLAST matches, further reflecting the divergent nature of their expression profiles.

**Figure 6.**
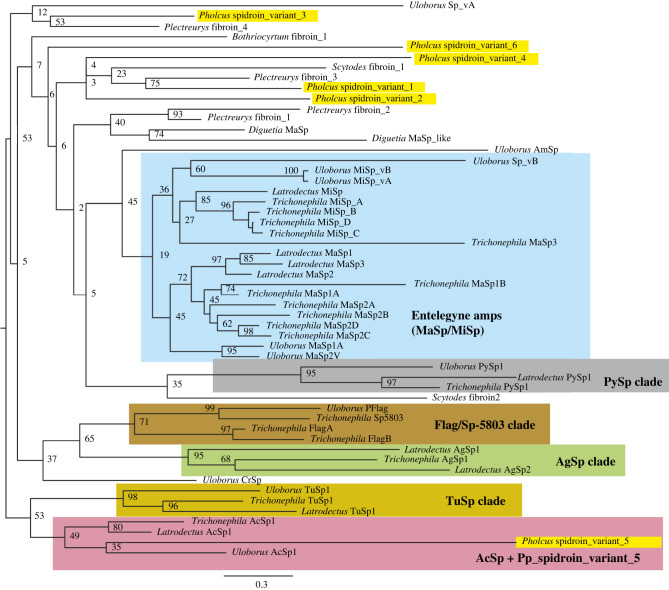
Spidroin phylogeny. Combined N- and C-terminal domain RaxML best tree, bootstrap values at nodes. Species from which spidroins are derived are labelled by their genus with species origin as follows: *Pholcus* = *P. phalangiodes*; *Bothriocyrtum* = *B. californicum* (mygalomorph); *Uloborus* = *U. diversus*; *Trichonephila* = *T. clavipes*; *Latrodectus* = *L. hesperus*; *Diguetia* = *D. canites*; *Plectreurys* = *P. tristis*; *Scytodes* = *S. thoracica*; *Pholcus* sequences highlighted in bright yellow.

The phylogenetic tree of *Pholcus* spidroins placed sequences of functionally defined spidroins from entelegyne species in their respective clades, according to gland of primary expression (MaSp, MiSp, TuSp, AcSp, Flag, AgSp, etc) (figure 6, electronic supplementary material, figure S6). However, *Pholcus* sequences did not group with these previously defined clades (except for spidroin 5, which was found nested in the AcSp clade in the ML but not the Bayesian analysis). Instead *Pholcus* spidroins displayed closer affinities to spidroins from other Synspermiata species, particularly those characterized (but non-annotated) from *Plectreurys tristis* with moderate to high support. Moreover, the deeper relationships between supported entelegyne spidroin clades to each other, as well as to non-entelegyne spidroins were not well supported.

## 4. Discussion

### 4.1. Viscid silk as softened piriform silk

Our results demonstrate an intriguing case where adhesive silks with distinct functions evolved from the same homologous basic type of silk gland, facilitating ecological versatility. Thread anchorage and prey trapping are functions with conflicting demands on glue properties. While a thread anchor must form a durable bonding, a prey trap must be permanently sticky and form only a temporary bonding.

In the first instance, the glue preferably has an initially low viscosity facilitating the spreading on the substrate surface and then rapidly solidifies to enhance cohesion. Such glues could also be used to immobilize prey, if directly applied on the prey. Our results suggest that the wrapping silk of *P. phalangioides* originates from the same gland that also produces the thread anchorages. This gland has been shown to exhibit histochemical characteristics similar to the piriform glands of other spiders [32]. Interestingly, both the involved piriform gland and the gland spigot are highly enlarged and can discharge much higher volumes of secretion than a typical piriform gland. While this is clearly not advantageous for thread anchoring (see Wolff [15]), it probably has evolved as an adaptation to the specific hunting technique. A similar case was found in gnaphosid spiders, which apply piriform silk to immobilize hazardous prey [53]. This change in piriform gland function was followed by an enlargement of the glands and spigots, too. Furthermore, in both Pholcidae and Gnaphosidae piriform silk spigot openings are widened to expel large amounts of secretion, and seem elastic and closed in rest. This may prevent water evaporation from the duct opening, which would harden the secretion and clog the spigot if not in use. However, gnaphosids do not add additional fibres to these capture lines, but instead their piriform silk retained the ancestral two compound composition (fibre + glue). In contrast to Pholcidae, the Gnaphosidae lost the ability to spin thread anchors and draglines. Thus, while the biological function of the piriform glands was changed in the Gnaphosidae, it was extended in the Pholcidae.

In contrast to the production of robust anchors and the agglutination of prey body parts, the glue for a permanent predatory snare needs to retain its stickiness, whereas a durable strong bonding is less important [54], as a spider will typically approach the trapped prey and subdue it using legs or silk wraps and, finally, venom [55]. To remain sticky, the material must stay soft, i.e. with an elastic modulus under 100 kPa (Dahlquist criterion, [56,57]). In the viscid silk of orb weavers (Araneidae) and cobweb spiders (Theridiidae) this is achieved by a high concentration of hygroscopic salts that keep the secretion highly hydrated, even in dry environments [9,29,30,58,59]. The hygroscopic salts also sequester water away from the glue–substrate interface, which enhances adhesion even in wet conditions where artificial pressure sensitive adhesives fail [60]. In the gumfoots of *P. phalangioides* a high concentration of betaine was previously found, which may fulfil the function of silk plasticization [9]. Here, Raman spectroscopy confirmed that the betaine is predominantly found in the viscid glue of the gumfoot and neither in the anchor nor in the thread.

The structure of the viscid silk was found remarkably similar to its araneid analogue [58]: when dried down in the vacuum of the scanning electron microscope there is a small solid granule and the remnants of the droplet exhibits salt deposits (figure 2*g*). The transcriptomes of the dissected glands showed highly contrasting spidroin expression profiles, with multiple spidroins being expressed in the large piriform gland, but only comparably small amounts in the small piriform glands. This is comparable to the expression profiles of the viscid glue glands in the distantly related Theridiidae that exhibit highly reduced spidroin expression but elevated expression of non-spidroin proteins and gland-specific peptides (so-called aggregate silk factors, AgSFs, and aqueous glue droplet peptides or spider-coating peptides, SCPs) compared with the fibre producing glands [61]. These similarities suggest a common composition–function correlation across convergently evolved viscid silk.

The Raman spectrum of the viscid silk showed a reduced amide III peak relative to the spectrum of the anchor glue. This peak can indicate crystalline (beta-sheet) secondary structures in the proteins [62], i.e. a reduced peak is consistent with a softer material.

We conclude that in the Pholcidae, the morphological differentiation of piriform glands facilitated the serving of two disparate functions. By a disparate change of gland size, the increase of the ejection duct and spigot diameter, and an increase of salt content in one of the gland products, the piriform silk of pholcids could additionally expand its applicability. This may have contributed to the remarkable ecological success of these spiders.

### 4.2. Spidroin evolution took a hereto unknown route in early diverging spider lineages

Pholcus spidroin 1, 5 and 3 were most highly expressed in the large piriform gland and may be the major protein components of this gland's adhesive secretions. In the transcriptome of the large piriform gland we, further, identified spidroin 6 that seems specific to this gland but, despite containing a complete coding sequence, contained no recognizable C-terminal domain. A case of a spidroin with a lost C-terminus has been documented before [63], and given the role of the C-termini in fibre formation [64], suggests such a spidroin may contribute to non-fibrous secretions. A caveat of our study is that no transcriptome of the ampullate and aciniform glands were obtained, inhibiting a complete functional annotation of all identified spidroins.

The comparison of *Pholcus* spidroin terminal domains with those that are functionally annotated from entelegyne (especially orb-weaving) spiders did not show them as belonging to known gland-specific spidroin classes (such as MaSp or PySp). This probably reflects the extremely long history since these species shared a common ancestor (over 300 million years [65]) with highly diverging spidroin evolution in different evolutionary lineages (potentially including changes in gland expression patterns of orthologous spidroin genes), reducing the phylogenetic signal in these sequences. Unfortunately, within spidroin genes there are no other domains than the termini that are evolutionarily more conserved, apparently hindering the inference of spidroin gene homology across such distant lineages. The limited functional characterization of non-entelegyne spidroins further hinders our ability to infer evolutionary transitions from one silk type to another. However, the distant relationship between *Pholcus* spidroins involved in their gluey secretions and those produced by theridiids (AgSp), in addition to the distant relationship of entelegyne piriform spidroins to those expressed in *Pholcus* piriform glands, suggest multiple routes to producing these materials through the co-option of distantly related spidroin paralogs. Clearly, more studies on gland-specific expression patterns are needed for early branching araneomorph lineages, such as Synspermiata, to enhance our understanding on the evolution of spidroin functions, and to correlate protein sequence signatures with analogous silk material properties across disparate evolutionary lineages.

### 4.3. From gumfoots to aerial traps

Our results suggest that in pholcids gumfoot threads evolved from silk anchors. In spiders, the spinning of silk anchors is relatively fast and requires the piriform glands to be rapidly activated and deactivated. This process is not without errors: In many spider species it has been observed that occasionally bundles of piriform silk and glue are attached to the emerging dragline for up to several millimetres (J.O.W., personal. observation). Depending on how fast the glue mass dries such ‘extended’ silk anchors could serve as a trap for running prey. A softening of the glue mass for an enhancement of this function may require a functional diversification of the piriform gland array as the original function—providing a robust thread anchorage—would be compromised by an increase of glue softness. It is possible that in pholcids the evolution of viscid silk was facilitated by the enlargement of one piriform gland that initially evolved as an adaptation to wrap attack behaviour. In a subsequent evolutionary stage, the viscid silk coating was also applied to aerial lines, as found in the pholcid genus *Modisimus* [66]. These pholcids construct aerial sheet webs resembling those of some Araneoidea [67]. This example shows one of multiple pathways, of how aerial sticky webs as adaptations to capture flying prey could have evolved.

Notably, such an evolutionary scenario as inferred above for the Pholcidae is in stark contrast to the strikingly analogous snares of cobweb spiders (Theridiidae) that have been hypothesized to have evolved from the capture spiral of orb webs [68–70]. We propose here that wrap attacks were a unifying key to the evolution of viscid silk in both groups, as it may be correlated with selective benefits of enlarged glands expelling large amounts of glue. These amounts are required to form droplets of a significant volume to generate a contact area with the prey that is suitable for retention. In the Araneoidea it was hypothesized that aggregate glands evolved from paracribellar or modified aciniform glands, both of which produce solid fibres [71,72]. However, the molecular mechanisms of this radical transition is largely unclear and difficult to explain [23]. More recently, it was suggested that the evolution of viscid silk and the loss of cribellar silk were separate events, because no sequences resembling proteins typical for aggregate secretions were found in the transcriptome of cribellate orb weavers [72], leaving the evolutionary origins of the ecologically impactful araneoid viscid silk enigmatic.

## 5. Conclusion

Here, by the intriguing case of the cosmopolitan pholcid spiders, it was demonstrated for the first time, how generalist homologous silk glands through specialization evolved contrasting properties of either solidifying or viscid adhesives. Using an interdisciplinary analytical approach, we uncovered evidence that these gland specializations correlated with a radical change in the secretion profiles, with the gland producing solidifying glues being heavily based on the expression of modified spidroins, and the glands producing viscid adhesives having almost completely downregulated spidroin expression, and highly upregulated organic salt production. As a result, *P. phalangioides* is equipped with a versatile machinery to capture prey, allowing it to exploit an enormous range of prey resources. This is not only an interesting case that permits the inference of the evolution of biological materials with profound ecological consequences, but by exhibiting these properties, pholcid silk glands could also inform the biomimetic design of novel adhesives based on biodegradable and biocompatible biopolymers with tailorable mechanical properties.

## Data Availability

Data are reported in the paper and electronic supplemental material. The raw transcriptomes are available from the NCBI SRA database: accession number PRJNA1096818. The data are provided in electronic supplementary material [73].

## References

[1] Foelix RF. 2011 Biology of spiders, 3rd edn. Oxford, NY: Oxford University Press.

[2] Eberhard W. 2020 Spider webs: behavior, function, and evolution. Chicago, IL: University of Chicago Press.

[3] Blamires SJ, Zhang S, Tso I-M. 2017 Webs: diversity, structure and function. In Behaviour and ecology of spiders: contributions from the Neotropical region (eds C Viera, MO. Gonzaga), pp. 137-164. Berlin, Germany: Springer.

[4] Wolff JO et al. 2019 Evolution of aerial spider webs coincided with repeated structural optimization of silk anchorages. Evolution **73**, 2122-2134. (10.1111/evo.13834) 31441504

[5] Bond JE, Opell BD. 1998 Testing adaptive radiation and key innovation hypotheses in spiders. Evol. Dev. **52**, 403-414. (10.2307/2411077) 28568335

[6] Liu J, May-Collado LJ, Pekár S, Agnarsson I. 2016 A revised and dated phylogeny of cobweb spiders (Araneae, Araneoidea, Theridiidae): a predatory Cretaceous lineage diversifying in the era of the ants (Hymenoptera, Formicidae). Mol. Phylogenet. Evol. **94**, 658-675. (10.1016/j.ympev.2015.09.023) 26454029

[7] Piorkowski D, Blackledge TA. 2017 Punctuated evolution of viscid silk in spider orb webs supported by mechanical behavior of wet cribellate silk. Sci. Nat. **104**, 1-12. (10.1007/s00114-017-1489-x) 28752413

[8] Blamires SJ. 2024 Grand challenges in arachnid genetics and biomaterials. Front. Arachnid Sci. **3**, 1356170. (10.3389/frchs.2024.1356170)

[9] Wolff JO, Cherry BR, Yarger JL, Adler L, Thomas DS, Hook JM, Blamires SJ. 2023 Organic salt composition of pressure sensitive adhesives produced by spiders. Front. Ecol. Evol. **11**, 1123614. (10.3389/fevo.2023.1123614)

[10] Japyassú HF, Macagnan CR. 2004 Fishing for prey: the evolution of a new predatory tactic among spiders (Araneae, Pholcidae). Revista de Etologia **6**, 79-94.

[11] Benjamin SP, Zschokke S. 2002 Untangling the tangle-web: web construction behavior of the comb-footed spider *Steatoda triangulosa* and comments on phylogenetic implications (Araneae: Theridiidae). J. Insect Behav. **15**, 791-809. (10.1023/A:1021175507377)

[12] Escalante I, Masís-Calvo M. 2014 The absence of gumfoot threads in webs of early juveniles and adult males of *Physocyclus globosu*s (Pholcidae) is not associated with spigot morphology. Arachnology **16**, 214-218. (10.13156/arac.2014.16.6.214)

[13] Argintean S, Chen J, Kim M, Moore A. 2006 Resilient silk captures prey in black widow cobwebs. Appl. Phys. A **82**, 235-241. (10.1007/s00339-005-3430-y)

[14] Sahni V, Harris J, Blackledge TA, Dhinojwala A. 2012 Cobweb-weaving spiders produce different attachment discs for locomotion and prey capture. Nat. Commun. **3**, 1-7. (10.1038/Ncomms2099) 23033082

[15] Wolff JO. 2017 Structural effects of glue application in spiders – what can we learn from silk anchors? In Bio-inspired structured adhesives (eds L Xue, L Heepe, SN Gorb), pp. 63-80. Dordrecht, The Netherlands: Springer Science+Business Media.

[16] Barrantes G, Eberhard WG. 2007 The evolution of prey-wrapping behaviour in spiders. J. Nat. Hist. **41**, 1631-1658. (10.1080/00222930701464364)

[17] Escalante I. 2015 Predatory behaviour is plastic according to prey difficulty in naïve spiderlings. J. Insect Behav. **28**, 635-650. (10.1007/s10905-015-9530-4)

[18] Kirchner W, Opderbeck M. 1990 Beuteerwerb, Giftwirkung und Nahrungsaufnahme bei der Zitterspinne *Pholcus phalangioides* (Araneae, Pholcidae). Verhandlungen des Naturwissenschaftlichen Vereins in Hamburg **31**, 15-45.

[19] Eberhard WG. 1992 Notes on the ecology and behaviour of Physocyclus globosus (Araneae, Pholcidae). Bull. Br. Arachnol. Soc. **9**, 38-42.

[20] Jackson RR, Brassington RJ. 1987 The biology of *Pholcus phalangioides* (Araneae, Pholcidae): predatory versatility, araneophagy and aggressive mimicry. J. Zool. **211**, 227-238. (10.1111/j.1469-7998.1987.tb01531.x)

[21] Ackermann G. 2012 *Lepidodactylus lugubris* (Squamata: Gekkonidae) als Beute von *Pholcus phalangioides* (Araneae: Pholcidae). Arachnologische Mitteilungen **44**, 14-16. (10.5431/aramit4404)

[22] O'Shea M, Kelly K. 2017 Predation on a weasel skink (*Saproscincus mustelinus*) (Squamata: Scincidae: Lygosominae) by a redback spider (*Latrodectus hasselti*) (Araneae: Araneomorpha: Theridiidae), with a review of other *Latrodectus* predation events involving squamates. Herpetofauna **44**, 49-55.

[23] Townley MA, Tillinghast EK. 2013 Aggregate silk gland secretions of araneoid spiders. In Spider ecophysiology, pp. 283-302. Berlin, Germany: Springer.

[24] Collin MA, Clarke TH, Ayoub NA, Hayashi CY. 2016 Evidence from multiple species that spider silk glue component ASG2 is a spidroin. Sci. Rep. **6**, 21589. (10.1038/srep21589) 26875681 PMC4753498

[25] Ayoub NA et al. 2023 Orb weaver aggregate glue protein composition as a mechanism for rapid evolution of material properties. Front. Ecol. Evol. **11**, 1099481. (10.3389/fevo.2023.1099481)

[26] Vollrath F, Tillinghast E. 1991 Glycoprotein glue beneath a spider web's aqueous coat. Naturwissenschaften **78**, 557-559. (10.1007/BF01134447)

[27] Vollrath F, Fairbrother WJ, Williams RJ, Tillinghast EK, Bernstein DT, Gallagher KS, Townley MA. 1990 Compounds in the droplets of the orb spider's viscid spiral. Nat. Cell Biol. **345**, 526-528.

[28] Sahni V, Miyoshi T, Chen K, Jain D, Blamires SJ, Blackledge TA, Dhinojwala A. 2014 Direct solvation of glycoproteins by salts in spider silk glues enhances adhesion and helps to explain the evolution of modern spider orb webs. Biomacromolecules **15**, 1225-1232. (10.1021/bm401800y) 24588057

[29] Sahni V, Blackledge TA, Dhinojwala A. 2011 Changes in the adhesive properties of spider aggregate glue during the evolution of cobwebs. Sci. Rep. **1**, 41. (10.1038/Srep00041) 22355560 PMC3216528

[30] Sahni V, Blackledge TA, Dhinojwala A. 2010 Viscoelastic solids explain spider web stickiness. Nat. Commun. **1**, 1-4. (10.1038/ncomms1019) 20975677

[31] Kirchner VW. 1986 Das Netz der Zitterspinne (*Pholcus phalangioides* Fuesslin)(Araneae: Pholcidae). Zool. Anz. **216**, 151-169.

[32] Kovoor J. 1986 Affinités de quelques Pholcidae (Araneae) décelables d'aprés les caractéres de l'appareil séricigéne. Mémoires de la Société Entomologique de Belgique **33**, 111-118.

[33] Apstein C. 1889 Bau und Funktion der Spinndrüsen der Araneida. Arch. Naturg. **55**, 29-74.

[34] Platnick NI, Coddington JA, Forster RR, Griswold CE. 1991 Spinneret morphology and the phylogeny of haplogyne spiders (Araneae, Araneomorphae). Am. Mus. Novit. **3016**, 1-73.

[35] Wolff JO, Michalik P, Ravelo AM, Herberstein ME, Ramírez MJ. 2021 Evolution of silk anchor structure as the joint effect of spinning behavior and spinneret morphology. Integr. Comp. Biol. **61**, 1411-1431. (10.1093/icb/icab003) 33616646

[36] Kovoor J, Zylberberg L. 1980 Fine-structural aspects of silk secretion in a spider (*Araneus diadematus*). 1. Elaboration in the pyriform glands. Tissue Cell **12**, 547-556. (10.1016/0040-8166(80)90044-0) 7434338

[37] Kovoor J, Zylberberg L. 1982 Fine-Structural Aspects of Silk Secretion in a Spider. 2. Conduction in the pyriform glands. Tissue Cell **14**, 519-530. (10.1016/0040-8166(82)90044-1) 6890724

[38] Wolff JO, Grawe I, Wirth M, Karstedt A, Gorb SN. 2015 Spider's super-glue: thread anchors are composite adhesives with synergistic hierarchical organization. Soft Matter **11**, 2394-2403. (10.1039/C4SM02130D) 25672841

[39] Schütt K. 1996 Wie Spinnen ihre Netze befestigen. Mikrokosmos **85**, 274-278.

[40] Wirth M, Wolff JO, Appel E, Gorb SN. 2019 Ultrastructure of spider thread anchorages. J. Morphol. **280**, 534-543. (10.1002/jmor.20962) 30791126

[41] Schneider CA, Rasband WS, Eliceiri KW. 2012 NIH Image to ImageJ: 25 years of image analysis. Nat. Methods **9**, 671-675. (10.1038/nmeth.2089) 22930834 PMC5554542

[42] Řezáč M, Krejčí T, Goodacre SL, Haddad CR, Řezáčová V. 2017 Morphological and functional diversity of minor ampullate glands in spiders from the superfamily Amaurobioidea (Entelegynae: RTA clade). J. Arachnol. **45**, 198-209. (10.1636/JoA-16-010-Rezak.1)

[43] Wolff JO. 2020 The evolution of dragline initiation in spiders: multiple transitions from multi-to single-gland usage. Diversity **12**, 4. (10.3390/d12010004)

[44] Blamires SJ, Nobbs M, Wolff JO, Heu C. 2022 Nutritionally induced nanoscale variations in spider silk structural and mechanical properties. J. Mech. Behav. Biomed. **125**, 104873. (10.1016/j.jmbbm.2021.104873) 34653899

[45] Heu C, Berquand A, Elie-Caille C, Nicod L. 2012 Glyphosate-induced stiffening of HaCaT keratinocytes, a Peak Force Tapping study on living cells. J. Struct. Biol. **178**, 1-7. (10.1016/j.jsb.2012.02.007) 22369932

[46] Kaemmer SB. 2011 Introduction to Bruker's ScanAsyst and PeakForce Tapping AFM Technology. Bruker Application Note **133**, 1-12.

[47] Strickland M, Tudorica V, Řezáč M, Thomas NR, Goodacre SL. 2018 Conservation of a pH-sensitive structure in the C-terminal region of spider silk extends across the entire silk gene family. Heredity **120**, 574-580. (10.1038/s41437-018-0050-9) 29445119 PMC5943517

[48] Eberhard WG. 2010 Possible functional significance of spigot placement on the spinnerets of spiders. J. Arachnol. **38**, 407-414. (10.1636/B09-97.1)

[49] Frutiger M, Kropf C. 2019 An anti-adhesive surface coating reduces adhesion during contact with cribellar threads in *Pholcus phalangioides* (Araneae, Pholcidae) but not in the web-owning spider *Uloborus plumipes* (Araneae, Uloboridae). *BioRxiv* 838250. (10.1101/838250)

[50] Neugirg BR, Koebley SR, Schniepp HC, Fery A. 2016 AFM-based mechanical characterization of single nanofibres. Nanoscale **8**, 8414-8426. (10.1039/C6NR00863A) 27055900

[51] Lefèvre T, Boudreault S, Cloutier C, Pézolet M. 2011 Diversity of molecular transformations involved in the formation of spider silks. J. Mol. Biol. **405**, 238-253. (10.1016/j.jmb.2010.10.052) 21050860

[52] Jehlička J, Oren A, Vítek P. 2012 Use of Raman spectroscopy for identification of compatible solutes in halophilic bacteria. Extremophiles **16**, 507-514. (10.1007/s00792-012-0450-3) 22527044

[53] Wolff JO, Řezáč M, Krejčí T, Gorb SN. 2017 Hunting with sticky tape: functional shift in silk glands of araneophagous ground spiders (Gnaphosidae). J. Exp. Biol. **220**, 2250-2259. (10.1242/jeb.154682) 28615490

[54] Wolff JO, Gorb SN. 2016 Attachment structures and adhesive secretions in arachnids. Cham, Switzerland: Springer International Publishing.

[55] Eggs B, Wolff JO, Kuhn-Nentwig L, Gorb SN, Nentwig W. 2015 Hunting without a web: how lycosoid spiders subdue their prey. Ethology **121**, 1166-1177. (10.1111/eth.12432)

[56] Dahlquist C. 1959 An investigation into the nature of tack. Adhes. Age **2**, 25-29.

[57] Creton C. 2003 Pressure-sensitive adhesives: an introductory course. MRS Bull. **28**, 434-439. (10.1557/Mrs2003.124)

[58] Amarpuri G, Chaurasia V, Jain D, Blackledge TA, Dhinojwala A. 2015 Ubiquitous distribution of salts and proteins in spider glue enhances spider silk adhesion. Sci. Rep. **5**, 9030. (10.1038/srep09030) 25761668 PMC4357010

[59] Townley MA, Bernstein DT, Gallagher KS, Tillinghast EK. 1991 Comparative study of orb web hygroscopicity and adhesive spiral composition in three araneid spiders. J. Exp. Zool. Part A **259**, 154-165.

[60] Singla S, Amarpuri G, Dhopatkar N, Blackledge TA, Dhinojwala A. 2018 Hygroscopic compounds in spider aggregate glue remove interfacial water to maintain adhesion in humid conditions. Nat. Commun. **9**, 1890. (10.1038/s41467-018-04263-z) 29789602 PMC5964112

[61] Clarke TH, Garb JE, Haney RA, Chaw RC, Hayashi CY, Ayoub NA. 2017 Evolutionary shifts in gene expression decoupled from gene duplication across functionally distinct spider silk glands. Sci. Rep. **7**, 8393. (10.1038/s41598-017-07388-1) 28827773 PMC5566633

[62] Lefevre T, Paquet-Mercier F, Rioux-Dubé JF, Pézolet M. 2012 Structure of silk by Raman spectromicroscopy: from the spinning glands to the fibers. Biopolymers **97**, 322-336. (10.1002/bip.21712) 21882171

[63] Correa-Garhwal SM, Babb PL, Voight BF, Hayashi CY. 2021 Golden orb-weaving spider (*Trichonephila clavipes*) silk genes with sex-biased expression and atypical architectures. G3 **11**, jkaa039. 33561241 10.1093/g3journal/jkaa039 PMC8022711

[64] Hagn F, Eisoldt L, Hardy JG, Vendrely C, Coles M, Scheibel T, Kessler H. 2010 A conserved spider silk domain acts as a molecular switch that controls fibre assembly. Nature **465**, 239-242. (10.1038/nature08936) 20463741

[65] Magalhaes IL, Azevedo GH, Michalik P, Ramírez MJ. 2020 The fossil record of spiders revisited: implications for calibrating trees and evidence for a major faunal turnover since the Mesozoic. Biol. Rev. **95**, 184-217. (10.1111/brv.12559) 31713947

[66] Briceno RD. 1985 Sticky balls in webs of the spider *Modisimus* sp. (Araneae, Pholcidae). J. Arachnol. **13**, 7.

[67] Eberhard WG. 1992 Web construction by *Modisimus* sp. (Araneae, Pholcidae). J. Arachnol. **20**, 25-34.

[68] Eberhard WG, Barrantes G, Madrigal-Brenes R. 2008 Vestiges of an orb-weaving ancestor? The ‘biogenetic law’ and ontogenetic changes in the webs and building behavior of the black widow spider *Latrodectus geometricus* (Araneae Theridiidae). Ethol. Ecol. Evol. **20**, 211-244. (10.1080/08927014.2008.9522523)

[69] Blackledge TA, Scharff N, Coddington JA, Szüts T, Wenzel JW, Hayashi CY, Agnarsson I. 2009 Reconstructing web evolution and spider diversification in the molecular era. Proc. Natl Acad. Sci. USA **106**, 5229-5234. (10.1073/pnas.0901377106) 19289848 PMC2656561

[70] Bond JE, Garrison NL, Hamilton CA, Godwin RL, Hedin M, Agnarsson I. 2014 Phylogenomics resolves a spider backbone phylogeny and rejects a prevailing paradigm for orb web evolution. Curr. Biol. **24**, 1765-1771. (10.1016/j.cub.2014.06.034) 25042592

[71] Opell BD, Tran AM, Karinshak SE. 2011 Adhesive compatibility of cribellar and viscous prey capture threads and its implication for the evolution of orb-weaving spiders. J. Exp. Zool. A Ecol. Genet. Physiol. **315**, 376-384. (10.1002/jez.684) 21445988

[72] Correa-Garhwal SM, Baker RH, Clarke TH, Ayoub NA, Hayashi CY. 2022 The evolutionary history of cribellate orb-weaver capture thread spidroins. BMC Ecol. Evol. **22**, 1-16. (10.1186/s12862-021-01952-0) 35810286 PMC9270836

[73] Wolff JO et al. 2024 From fibres to adhesives: evolution of spider capture threads from web anchors by radical changes in silk gland function. *Figshare*. (10.6084/m9.figshare.c.7353450) PMC11289648 39081115

